# Attending to the Existential Experience in Oncology: Dignity and Meaning Amid Awareness of Death

**DOI:** 10.1200/GO.22.00038

**Published:** 2022-03-14

**Authors:** William E. Rosa, Harvey M. Chochinov, Nessa Coyle, Rachel A. Hadler, William S. Breitbart

**Affiliations:** ^1^ Department of Psychiatry and Behavioral Sciences, Memorial Sloan Kettering Cancer Center, New York, NY; ^2^ Department of Psychiatry, Max Rady College of Medicine, University of Manitoba, Manitoba, Canada; ^3^ CancerCare Manitoba Research Institute, Manitoba, Canada; ^4^ Ethics Committee, Memorial Sloan Kettering Cancer Center, New York, NY; ^5^ Department of Anesthesiology, University of Iowa Hospitals and Health Clinics, Iowa City, IA, USA

The COVID-19 pandemic has deeply challenged the integrity of the human spirit. Health systems worldwide have seen a marked escalation in health-related suffering and uncertainty about the future. Beyond these broader collective concerns, patients with cancer continue to confront treatment disruptions and increased isolation from support systems. Moral distress and burnout are leading to clinical staff exhaustion and depletion across settings. As cancer clinicians adapt to a rapidly changing world characterized by viral variants and global inequities, we call for a widespread commitment to recognize and prioritize the existential experience of all persons with cancer as a moral standard of care.

By *existential experience*, we mean the patient's fluctuating state of awareness related to their own mortality and death.^1^ This existential experience may shift between *existential suffering* (disturbance of soul and spirit) and *existential health* (wholeness and connectedness with self and others).^1^ Many cancer clinicians have witnessed firsthand the sting of the existential slap—a patient's emerging, yet sometimes sudden, awareness that death is an inevitability.^2^ Once acknowledged, they cannot unknow the fragile limits of their human form and distress often ensues. The ever-present threat of loss to personhood, health, relationships, and, ultimately, life is omnipresent in the cancer experience; acknowledging these existential concerns can support coping, autonomy, and existential health.^3^ Providing consistent existential care through humanistic responses to the patient's existential experience may help alleviate internal angst as one encounters disease transitions that increase proximity to death.

Interventions designed to enhance meaning and purpose have been empirically shown to decrease anxiety, depression, demoralization, and a desire for hastened death for patients with cancer-related existential suffering.^4^ By drawing on sources of meaning from the patient's narrative, we know that patients with life-threatening cancer possess the innate capacity to actively create their legacy and engage with life regardless of prognosis. Dignity enhancing approaches foster meaning making and generativity (eg, investment in loved ones who will be left behind) in relation to an individual's life, relationships, and values.^5^

Despite evidence that meaning-making improves the existential experience, it is unclear who owns the provision of existential care. The existential experience is typically underemphasized or entirely absent as a clinical priority. If included at all, medical and nursing curricula commonly address existential care under the umbrella of broader psychosocial phenomena without distinct attention to developing literacies in understanding and specifically addressing the existential experience. However—by way of contract—existential issues are innately personal and foundational to the cancer experience, hence underscoring a moral obligation that they be acknowledged and addressed.

Existential care is a core component of palliative care, and integrated palliative care is foundational to high-quality oncologic care.^6,7^ As palliative care models seek to relieve cancer-related suffering, how will oncology clinicians and systems prioritize existential literacy moving forward? Person-centered care is an illusion if frontline clinicians cannot or will not embed existential care principles throughout the relational aspects of cancer care. The very experience of living sees us all—patients and clinicians alike—grapple with existential issues. If clinicians are incapable of coming to terms with their own existential experience, being fully available to the existential experiences of patients is impossible. A common misnomer is that existential suffering is limited to end-of-life settings, but existential suffering may arise at any point along the cancer trajectory. Investments are needed to cultivate clinicians capable of forging human conversations and connections with patients and to approach the human dilemma of living and dying with compassion.^8^ Table 1 provides pragmatic approaches to promote existential literacy throughout oncology culture.

**TABLE 1:**
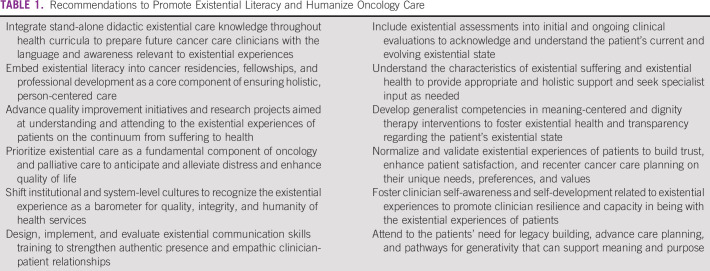
Recommendations to Promote Existential Literacy and Humanize Oncology Care

The pandemic has led to cumulative loss and mass bereavement on a global scale, heightening the public's collective death salience and existential terror and informing the need for existential maturity to mitigate mortality-related fears.^9^ When experiencing existential terror, humans may become more polarized and have diminished capacity for empathy.^10^ However, the hope inherent to existential care is one of creating a future in the face of uncertainty and the courage to do the hard work of living in the face of death.

Cancer treatment is a microcosm of the COVID-19 crisis: victories, losses, hopes, fears, uncertainty, and the potential for healing and cure. The pandemic has sparked urgency for all clinicians to build capacities to recognize and respond to the existential experiences of patients and to partner with patients in preserving the essence of who they are amid the unpredictability of cancer. Patients' existential concerns are ubiquitous and deserving of our attention. Indeed, it is time we call those concerns by name.
